# Predictors of mortality and poor outcome for patients with severe infectious encephalitis in the intensive care unit: a cross-sectional study

**DOI:** 10.1186/s12879-024-09312-1

**Published:** 2024-04-22

**Authors:** WenYan Zhao, YuLiang Zhou, YingYing Hu, WenJing Luo, Jing Wang, Hong Zhu, ZhiPeng Xu

**Affiliations:** 1 Department of Neuropsychology, Zhongnan Hospital of Wuhan University, Wuhan, People’s Republic of China; 2 Department of Neurology, General Hospital of Central Theater Command, Wuhan, People’s Republic of China

**Keywords:** Outcome, Encephalitis, Predictor, Intensive care units, Mortality

## Abstract

**Background:**

There are few thorough studies assessing predictors of severe encephalitis, despite the poor prognosis and high mortality associated with severe encephalitis. The study aims to evaluate the clinical predictors of mortality and poor outcomes at hospital discharge in patients with severe infectious encephalitis in intensive care units.

**Method:**

In two Chinese hospitals, a retrospective cohort study comprising 209 patients in intensive care units suffering from severe infectious encephalitis was carried out. Univariate and multivariate logistic regression analyses were used to identify the factors predicting mortality in all patients and poor outcomes in all survivors with severe infectious encephalitis.

**Results:**

In our cohort of 209 patients with severe encephalitis, 22 patients died, yielding a mortality rate of 10.5%. Cerebrospinal fluid pressure ≥ 400mmH_2_O (OR = 7.43), abnormal imaging (OR = 3.51), abnormal electroencephalogram (OR = 7.14), and number of rescues (OR = 1.12) were significantly associated with an increased risk of mortality in severe infectious encephalitis patients. Among the 187 survivors, 122 (65.2%) had favorable outcomes, defined as the modified Rankine Scale (mRS) score (0 ~ 3), and 65(34.8%) had poor outcomes (mRS scores 4 ~ 5). Age (OR = 1.02), number of rescues (OR = 1.43), and tubercular infection (OR = 10.77) were independent factors associated with poor outcomes at discharge in all survivors with severe infectious encephalitis.

**Conclusions:**

Multiple clinical, radiologic, and electrophysiological variables are independent predictive indicators for mortality and poor outcomes in patients with severe encephalitis in intensive care units. Identifying these outcome predictors early in patients with severe encephalitis may enable the implementation of appropriate medical treatment and help reduce mortality rates.

**Supplementary Information:**

The online version contains supplementary material available at 10.1186/s12879-024-09312-1.

## Background

A serious medical disorder called encephalitis is characterized by inflammation of the brain and resulting in neurological impairment. Encephalitis is a potentially fatal condition that affects 12.6 out of every 10,000 people each year [1]. It is primarily brought on by autoimmune and infectious diseases and is clinically characterized by fever, headache, seizures, and altered consciousness. However, the etiology is still unknown in 40–50% of encephalitis cases [2, 3].

Even with the advancements in encephalitis diagnosis and treatment, 7–18% of patients still die from the illness. The annual cost of hospitalizing patients with viral encephalitis in the United States is estimated to be between $350 million and $540 million [4]. Additionally, up to 56% of survivors have severe disabilities, indicating the substantial financial burden that long-term care and rehabilitation costs place on patients and their families. Currently, antimicrobial agents, anti-inflammatory drugs, steroids, intravenous immunoglobulin (IVIG), and supportive care are the only therapy options available for individuals with encephalitis [5]. Early diagnosis and treatment of encephalitis have the potential to enhance the prognosis of this serious disease.

Because of the potential seriousness and risk of unexpected death, many patients with severe encephalitis need to stay in intensive care units (ICUs) for an extended period of time [6]. Previous research has demonstrated that encephalitis patients’ poor outcomes were significantly predicted by admission to the intensive care unit (ICU) [7]. However, nothing is known regarding the short-term outcomes of patients with severe encephalitis discharged from intensive care units.

In this study, we specifically examined a population of severe infectious encephalitis patients to identify predictors of mortality and discharge outcomes. We aim to help with early identification and intervention of the disease, assist ICU teams in tailoring treatment plans and supportive care based on different outcomes, increase the possibility of brain functional recovery, and ultimately improve overall outcomes.

## Methods

### Patients and definitions

This study was approved by the hospital ethics committee on human research. The study sample consisted of patients who were clinically diagnosed with severe infectious encephalitis at Zhongnan Hospital of Wuhan University and General Hospital of Central Theater Command from 2003 to 2022. Encephalitis cases were retrospectively identified within two hospitals’ databases based on the ICD-10 diagnosis codes corresponding to encephalitis, which were confirmed by neurologists’ notes, laboratory results, and neuroimaging data. Encephalitis was diagnosed as altered mental status (altered level of consciousness or personality change) lasting more than 24 hours, with no identified alternative cause; associated with three or more of the following: fever, seizures, focal neurological signs, cerebrospinal fluid (CSF) white blood cell (WBC) count > 5/µl and suggestive magnetic resonance imaging (MRI) or electroencephalogram (EEG) findings [8].

Patients with infectious encephalitis who were continuously admitted to the neurology ICU were also included. Encephalitis patients have to be admitted to the ICU if they meet one of the following criteria: (a) severe neurological damage (at least two seizures or status epilepticus; Glasgow score < 13); (b) other organ failures (shock, respiratory distress syndrome, etc.); (c) behavior disorders preventing hospitalization in a standard unit [9]. The diagnosis of infectious encephalitis is based on the comprehensive judgment of medical history, physical examination, cerebrospinal fluid examination, pathogenic testing, imaging examination, and electroencephalogram.

Study exclusion criteria were as follows: (a) meningitis without clinical brain involvement, autoimmune encephalitis, and noninfectious CNS diseases [10]. (b) the length of stay in the ICU is less than 48 hours, as 48 hours was identified as the minimum length of stay in the ICU to exclude those encephalitis patients who had only transient critical care needs [11].

In addition, rescue in our study meant that the patient needed to maintain basic vital signs through cardiopulmonary resuscitation (CPR), fluid resuscitation, electrical defibrillation, cardioversion or defibrillation, the use of a vasopressor, and other rescue measures.

### Etiological diagnosis

Pathogens can be identified by CSF Gram staining, culture of bacteria, mycobacterial culture and acid-fast bacillus stain, cryptococcal antigen, fungal culture, and serology or CSF amplification of the viral genome by RT-PCR or PCR. Recently, metagenomic next-generation sequencing (NGS) of cerebrospinal fluid has been applied clinically due to its advantages of identifying a wide range of pathogens [12]. Until the year 2018, we identified pathogens based on clinical evaluation, imaging examination, cerebrospinal fluid examination, etc. This study identified a pathogen in 34 patients. Therefore, this study could not make an analysis based on the pathogen. However, in the supplemental table (named Table 4), we have included a column that shows the patients for whom a specific pathogen was identified and another column where the etiology was determined as bacterial, viral, fungal, and tuberculous based on clinical evaluation, imaging examination, cerebrospinal fluid examination, etc.

### Data collection

Demographic characteristics (age, sex), immunization state, history of disease, physical examination, onset time, hospitalized days, length of admission to ICU, days of critical condition, number of rescues, medication administration, and complication in all severe encephalitis patients were recorded, respectively. Clinical information included cerebrospinal fluid (CSF) profile (white blood cell count, red blood cell count, glucose, protein, opening pressure, culture/PCR data), electroencephalogram (EEG), and head MRI. A critical condition is defined as being seriously ill or injured and likely to die. Complications of encephalitis include pulmonary infection, epilepsy, gastrointestinal hemorrhage, electrolyte disturbance, hypoproteinemia, heart failure, renal failure, etc. The abnormal electroencephalogram includes: the basic rhythm and volatility of each district increases or decreases, the presence of focal or generalized slow waves, and epileptiform discharges [13]. Sagittal and axial T1-weighted and T2-weighted images, thin-section coronal fluid-attenuated inversion recovery (FLAIR) sequences, and susceptibility-weighted images were obtained for all patients. MRI abnormalities were evaluated as lobe lesions, increased T2 and FLAIR signals, and contrast enhancement [14].

The modified Rankin scale (mRS), a clinician-reported measure of global disability, is widely applied for evaluating patient outcomes and as an endpoint in randomized clinical trial and was used to assess the prognosis of survivors at discharge [15]. Neurological status was assessed with the modified Rankin scale (mRS) at the discharge point. In those patients who survived, a good outcome was defined as mRS scores 0 to 3 and a poor outcome as mRS scores 4 to 5. Mortality included patients who died after being placed in comfort care or secondary to a medical complication following treatment.

### Statistical analysis

Statistical analysis was performed using SPSS 25.0 window software (statistical package for the social sciences, SPSS Inc.) and GraphPad Prism 8.4 software. All continuous data with a skewed distribution were descriptively presented using the median (interquartile range) and categorical data using the count (percentage [%]). Differences between groups were assessed with the Mann-Whitney U test for different kinds of continuous variables and chi-square for categorical variables. Multivariable logistic regression analysis was used to explore the association of factors with mortality in all encephalitis patients and poor outcomes in those surviving hospital discharge. A *p*-value < 0.05 was considered statistically significant.

## Results

### Demography

From the encephalitis databases in the two hospitals, 209 of a total of 683 patients with severe infectious encephalitis met our inclusion criteria. The median age was 36 years (23.0–53.0). 135 of 209 patients (64.6%) were male (Table 1). Patients were admitted after a median of 4.0(2.0–7.0) days of illness, the total length of hospital stay was 17.0(9.5–30.5) days, median length of admission to ICU reached 6.0(3.0–13.0) days, median days critical condition was 9.0 (4.0-18.8), the numbers of rescue was 0.0(0.0–1.0) times, median days of advanced care was 6.0(3.0–12.0) and primary care was 12.0(6.0-22.5).

**Table 1:** Characteristics of the study population (*n* = 209)

Variables	Data
Demography data	
Mean age (years)	36.0(23.0,53.0)
Male (%)	135(64.6%)
Hospitalization data	
Onset time (days)	4.0(2.0,7.0)
Hospitalized days	17.0(9.5,30.5)
Admission to ICU (days)	6.0(3.0,13.0)
Days of critical condition (days)	9.0(4.0,18.8)
Number of rescuing	0.0(0.0,1.0)
Days of advanced care (days)	6.0(3.0,12.0)
Days of primary care (days)	12.0(6.0,22.5)
Clinical data	
Cerebrospinal fluid pressure ≥ 180mmH_2_O	109(52.4%)
Cerebrospinal fluid pressure ≥ 400mmH_2_O	32(15.4%)
White blood cell abnormality in CSF	137(65.9%)
Total protein abnormality in CSF	137(65.6%)
Abnormal imaging	78(38.6%)
Abnormal electroencephalogram	65(32.8%)
Complications	118(56.5%)
Causative agent	
Viral	147(70.3%)
Bacterial	36(17.2%)
Fungal	10(4.8%)
Tubercular	16(7.7%)

Categorical variables are shown as frequencies. Numerical variables are expressed as median (interquartile range). Reference interval: CSF WBC count: 0–10 × 10^6^/L; CSF protein level, 0.15–0.45 g/L;

### Investigations

Out of the 209 included patients, 209 had a lumbar puncture, 202 had an MRI and 198 had an EEG done. In CSF examination, 32 of 208 patients (15.4%) had cerebrospinal fluid pressure above 400 mmH_2_O, 137 of 208 patients (65.9%) had white blood cell abnormality, and 137 of 209 patients (65.6%) had total protein abnormality. An abnormal electroencephalogram was observed in 65 of 198 patients (32.8%). An abnormal imaging was observed in 78 of 202 patients (38.6%). Complication was observed in 118 of 209 patients (56.5%) with encephalitis. The causative agent of encephalitis included 147 patients (70.3%) with viral encephalitis, 36 patients (17.2%) with bacterial encephalitis, 10 patients (4.8%) with fungal encephalitis, and 16 patients (7.7%) with tubercular encephalitis.

### Predictors of mortality

In our patient cohort, 22 patients died, leading to an average mortality rate of 10.5%. There was a notable distinction between surviving and dead patients in hospitalized days, days of critical condition, days of primary care, number of rescues, cerebrospinal fluid pressure ≥ 180mmH_2_O, abnormal imaging, abnormal electroencephalogram, and complication (Table 2). Given the presence of interrelationships among predictors of variables associated with mortality, we first used univariate logistics regression to analyze the predictors of severe encephalitis death and selected appropriate variables according to the results of the univariate analysis to be included in the multivariate regression analysis model (Fig. A). Multivariate logistic regression models demonstrated that CSF pressure ≥ 400mmH_2_O(OR = 7.43), abnormal imaging (OR = 3.51), and abnormal electroencephalogram (OR = 7.14) and numbers of rescue (OR = 1.12) were associated with mortality in patients with severe infectious encephalitis.

**Table 2:** The difference between survivors and death patients

Factors	Survivors(*n* = 187)	Death(*n* = 22)	Z/χ^2^	*p*-value
Mean age (years)	36.0(23.0,53.0)	37.5(23.3,54.3)	-0.3	0.747
Male (%)	120(64.2%)	15(68.2%)	0.1	0.710
Onset time (days)	4.0(2.0,7.0)	4.0(1.8,7.5)	-0.4	0.657
Hospitalized days	19.0(10.0,32.0)	9.0(2.8,18.3)	-3.3	0.001*
Admission to ICU (days)	6.0(3.0,12.0)	7.0(2.5,15.5)	-0.5	0.588
Days of critical condition	10.0(5.0,20.3)	5.0(2.0,15.3)	-2.1	0.038*
Number of rescuing	0.0(0.0,0.0)	1.5(0.8,3.0)	-5.2	<0.001*
Days of advanced care	6.0(3.0,12.0)	7.5(2.8,7.5)	-0.7	0.507
Days of primary care	13.0(7.0,24.0)	1.0(1.0,7.0)	-3.1	0.002*
CSFP				
CSFP ≥ 180mmH_2_O	89(47.8%)	20(90.9%)	14.6	<0.001*
CSFP ≥ 400mmH_2_O	25(13.4%)	7(31.8%)	5.1	0.024*
White blood cell abnormality in CSF	124(66.3%)	13(61.9%)	0.2	0.686
Total protein abnormality in CSF	123(65.8%)	14(63.6%)	0.0	0.842
Abnormal imaging	63(34.8%)	14(70.0%)	9.4	0.002*
Abnormal electroencephalogram	52(29.2%)	13(65.0%)	10.4	0.001*
Complication	96(51.3%)	22(100.0%)	19.0	< 0.001*
Causative agent				
Viral	135(72.2%)	12(54.5%)	5.4	0.145
Bacterial	31(16.6%)	5(22.7%)		
Fungal	7(3.7%)	3(13.6%)		
Tubercular	14(7.5%)	2(9.1%)		

Categorical variables are shown as frequencies. Numerical variables are expressed as median (interquartile range). * *P* < 0.05

**Fig. A:**
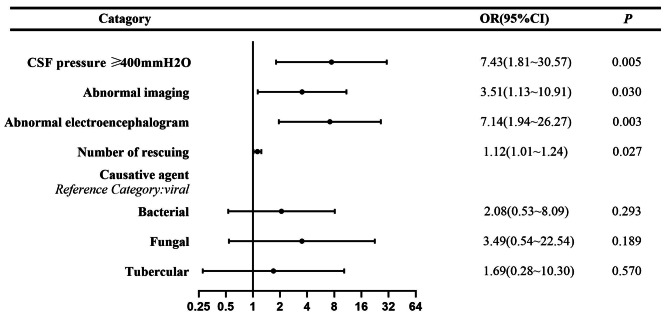
Forest plot of mortality risk prediction for severe infectious encephalitis in the ICU. We included plausible variables associated with death of severe infectious encephalitis in univariate logistics regression analysis into multivariate logistics regression analysis to obtain death predictors of severe infectious encephalitis. The variables on the right of the midline (OR = 1) and CIs not crossing the midline were independent predictors of severe encephalitis mortality in the ICU.

### Predictors of outcome among survivors

Of the surviving 187 patients, 122(65.2%) had good outcome (mRS scores 0–3) and 65(34.8%) had poor outcome (mRS scores 4–5). Advanced age, number of rescues, abnormal imaging, and species of causative agent are risk factors for poor prognosis (Table 3). We screened the predictors of poor prognosis according to univariate regression analysis and then performed multivariate retrospective analysis. It was found that advanced age (OR = 1.02), number of rescues (OR = 1.43), and tubercular infection (OR = 10.77) were independently associated with poor prognosis in survivors of severe infectious encephalitis at discharge (Fig. B).

**Table 3:** The difference between good prognosis and poor prognosis

Factors	mRS<3(*n* = 122)	3 ≤ mRS<6(*n* = 65)	Z/χ^2^	*p* value
Mean age (years)	33.0(22.0,50.0)	42.0(23.0,56.5)	-2.0	0.041*
Male (%)	80(65.6%)	40(61.5%)	0.3	0.584
Onset time (days)	3.5(2.0,7.0)	4.0(2.0,8.5)	-1.3	0.183
Hospitalized days	18.0(11.0,29)	19.0(8.5,48.0)	-0.4	0.706
Admission to ICU (days)	6.0(3.0,11.0)	6.0(2.0,18.5)	-0.9	0.386
Days of critical condition	8.0(4.0,14.0)	16.0(5.0,30.5)	-2.6	0.010*
Number of rescuing	0.0(0.0,0.0)	0.0(0.0,2.5)	-3.9	<0.001*
Days of advanced care	5.0(3.0,9.0)	8.0(3.0,20.0)	-2.0	0.044*
Days of primary care	14.0(7.0,21.5)	12.0(6.0,30.5)	-0.3	0.771
CSFP				
CSFP ≥ 180mmH_2_O	54(44.3%)	35(54.7%)	1.8	0.175
CSFP ≥ 400mmH_2_O	14(11.5%)	11(17.2%)	1.2	0.278
White blood cell in CSF>10/uL	80(65.6%)	44(67.7%)	0.1	0.770
White blood cell in CSF>500/uL	20(16.4%)	5(7.7%)	2.8	0.096
Total protein in CSF>0.4 g/L	81(66.4%)	42(64.6%)	0.1	0.807
Total protein in CSF>1 g/L	31(25.4%)	23(35.4%)	2.1	0.152
Abnormal imaging	34(28.6%)	29(46.8%)	6.0	0.015*
Abnormal electroencephalogram	35(29.9%)	17(27.9%)	0.1	0.776
Complication	57(46.7%)	39(60.0%)	3.0	0.084
Causative agent				
Viral	94(77.0%)	41(63.1%)	15.0	0.002*
Bacterial	22(18.0%)	9(13.8%)		
Fungal	3(2.5%)	4(6.2%)		
Tubercular	3(2.5%)	11(16.9%)		

Categorical variables are shown as frequencies. Numerical variables are expressed as median (interquartile range). * *P* < 0.05

**Fig. B:**
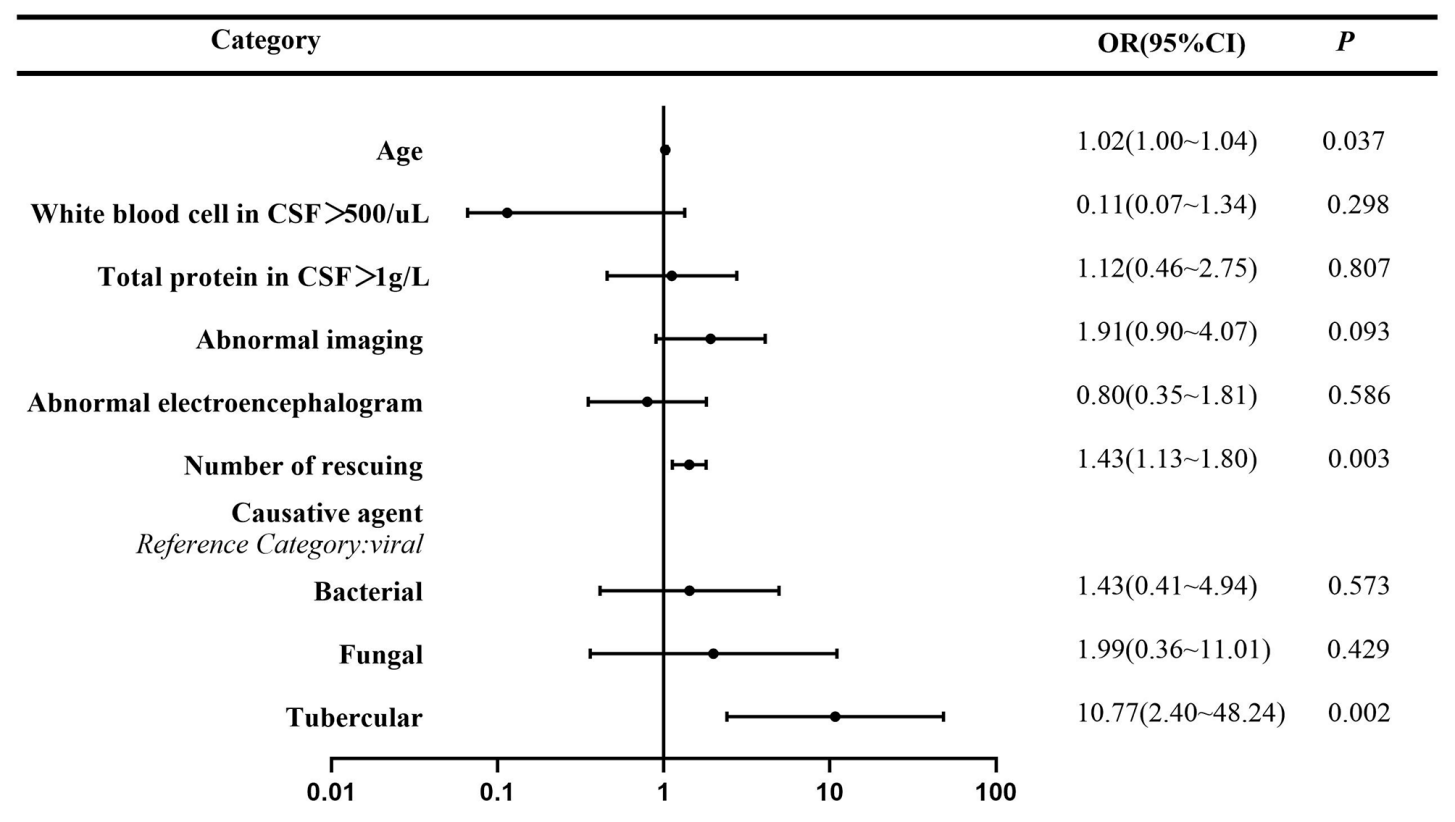
Forest plot of poor prognosis prediction for severe infectious encephalitis in the ICU

## Discussion

Encephalitis is a global health issue with a high mortality rate. Survivors of this condition face various complications, such as cognitive impairment, behavior disorder, and activity disorder. In this study, we retrospectively analyzed patients with severe infectious encephalitis. Our results indicate that neuroimaging, particularly MRI, cerebrospinal fluid (CSF) analysis, and electroencephalogram (EEG) examination, play a crucial role in diagnosing encephalitis.

### Factors related to mortality in patients with severe infectious encephalitis

Our study revealed several negative predictors of mortality in our patient cohort, including CSF pressure ≥ 400mmH_2_O, abnormal imaging, abnormal electroencephalogram, and the number of rescues.

Our findings suggest that a CSF pressure of ≥ 400mmH_2_O is associated with mortality. These findings are consistent with earlier research [16], which suggests that increased CSF pressure can lead to the compression of brain tissue, displacement of brain structures, the development of hydrocephalus, brain herniation, and a restricted blood supply to the brain, ultimately resulting in irreversible brain damage and death. Increased CSF pressure may indicate a more severe inflammatory response, potentially leading to greater cellular damage and neurological deficits. Therefore, increased CSF pressure serves as a late-stage indicator of a patient’s condition and prognosis.

In this study, 78(38.6%) patients had abnormal imaging involving cerebral lesions, which showed increased T2 and FLAIR signals, contrast enhancement, and lobe lesions. The mortality rate of encephalitis (10.5%) in the present study is consistent with worldwide encephalitis mortality (5–15%) [1]. Patients with encephalitis who have restricted diffusion lesions in MRI imaging fare worse clinically than those who do not. Moreover, patients with a greater extent of image abnormality have a poorer outcome [17, 18]. A multicenter cohort study on herpes simplex encephalitis revealed that extensive lesions in over three lobes on MRI upon admission were indicative of poor outcomes (such as moderate to severe disability and death) [19].

Out of the 209 patients, 65 (32.8%) had abnormal EEG. The possible mechanism involves neuronal necrosis and inflammatory cell infiltration resulting from infectious encephalitis. This, in turn, leads to abnormal firing of neurons [20]. EEG findings in encephalitis patients correlate with disease severity and have prognostic significance. Studies have demonstrated that worsening EEG findings indicate unfavorable outcomes [21]. EEG in the early stages of encephalitis is important for both diagnosis and prognosis. When combined with the overall severity of the clinical picture, it facilitates outcome evaluation and diagnosis [22].

Based on multivariate analysis, patients with severe infectious encephalitis who require many rescues are more likely to have additional concomitant illnesses, including dementia, brain damage, disability, or seizures. These additional concomitant illnesses prevent them from recovering functionally. According to our research, the number of rescues is a critical indicator of clinical severity and is associated with a fatal outcome in encephalitis. Given the unavoidable need for rescues, the ICU team must strive to optimize the rescue process and minimize adverse complications to enhance the prognosis.

### Prognostic factors for survivors of severe encephalitis

In our study, we found that 34.6% of patients with severe infectious encephalitis had poor outcomes. This study suggests that advanced age, the number of rescues, and tuberculosis infection were independently associated with poor outcomes for survivors at discharge in patients with severe infectious encephalitis.

An earlier study established that age was a risk factor that was independently linked to a poor prognosis for encephalitis patients during their hospital stay [23]. Singh’s research also reported a similar finding through the use of multivariate regression analysis. It revealed that individuals aged 65 years or older have a worse prognosis in cases of acute encephalitis [2]. However, previous studies in adult patients found no relationship between the age of patients with encephalitis and prognosis, suggesting that this parameter may only be relevant in studies including both adults and children [24, 25].

By analyzing the characteristics of causative agents that cause encephalitis, tuberculosis infection was identified as an independent predictor of poor prognosis, with viral infection as the reference category. A study conducted in France revealed that tuberculous encephalitis had a considerably higher mortality rate compared to other forms of encephalitis. Additionally, it was found to cause persistent neurological symptoms upon discharge, particularly movement disorders [26]. A prospective multicentre observational study has shown that viral encephalitis independently predicts 6-month mortality [27]. This is different from our findings and may be related to the fact that the proportion of encephalitis caused by L. monocytogenes and VZV increased with age, the latter becoming the main cause of infectious encephalitis after 80 years of age, and the proportion of TBE and M. tuberculosis encephalitis decreased with age [27].

### Limitations

This study has certain limitations. Firstly, our study involved two centers and other centers need to reproduce similar results to confirm them. Secondly, the design of our study did not allow long-term follow-up in survivors. Therefore, the long-term morbidity in survivors may have been underestimated. Thirdly, 48 hours is not enough to complete imaging, EEG, and lumbar puncture examinations; therefore, we did not include these cases that were only clinically considered to have encephalitis. Finally, since the past few decades, there has been a substantial improvement in the diagnostic procedures used to identify infections causing encephalitis, and this study is retrospective for a long period. Only a small number of etiological diagnoses were found in our retrospective analysis, and the test procedures employed to identify viral and bacterial pathogens altered throughout the investigation. This may have affected our findings. Future prospective multicenter studies are necessary to assess whether the prevention and management of these complications can improve outcomes in patients with severe encephalitis.

### Electronic supplementary material

Below is the link to the electronic supplementary material.

Supplementary Material 1

Supplementary Material 2

## Data Availability

The datasets used and/or analyzed during the current study are available from the corresponding author upon reasonable request.

## Abbreviations

IVIG: Intravenous Immunoglobulin
ICU: Intensive Care Unit
CPR: Cardiopulmonary Resucitation
PCR: Polymerase Chain Reaction
NGS: Next Generation Sequencing
CSF: Cerebrospinal Fluid
EEG: Electroencephalogram
FLAIR: Fluid-Attenuated Inversion Recovery
MRS: Modified Rankin Scale
