# Supplementary material for: Predictors of mortality and poor outcome for patients with severe infectious encephalitis in the intensive care unit: a cross-sectional study
Source: BMC Infect Dis. 2024 Apr 22;24:421. doi: 10.1186/s12879-024-09312-1 (PMC11034050; doi:10.1186/s12879-024-09312-1)
Supplement: Supplementary file 1 — Supplementary Material 1 [file 12879_2024_9312_MOESM1_ESM.doc]

**Table 4** Original data of 209 patients with severe infectious encephalitis

| Patient number | Sex：1.male, 2.female | Age | Onset time (days) | Cerebrospinal fluid pressure(mmH2O) | White blood cell in CSF(uL) | Total protein in CSF(g/L) | admission date | Abnormal imaging:1.Yes,0.No | Abnormal electroencephalogram:1.Yes,0.No | Causative agent:1.Viral,2.Bacterial,3.Fungal,4.Tubercular | Proven aetiology | viral encephalitis based on clinical:1.Yes,0.No | Complication:1.Yes,0.No | Death:1.Yes,0.No | Number of rescuing | Days of critical condition (days) | Admission to ICU (days) | Days of advanced care (days) | Days of primary care (days) | Hospitalized days | mRS Score>3:1.Yes,0.No |
| --- | --- | --- | --- | --- | --- | --- | --- | --- | --- | --- | --- | --- | --- | --- | --- | --- | --- | --- | --- | --- | --- |
| 1 | 1 | 59 | 7 | 80 | 9 | 0.15 | 2003/5/7 | 1 | 0 | 1 |  | 1 | 1 | 0 | 6 | 16 | 11 | 11 | 0 | 11 | 1 |
| 2 | 2 | 18 | 3 | 170 | 0 | 0.29 | 2003/5/19 | 0 | 0 | 1 |  | 1 | 0 | 0 | 0 | 6 | 2 | 2 | 12 | 13 | 1 |
| 3 | 2 | 20 | 4 | 155 | 50 | 0.13 | 2003/9/17 | 0 | 0 | 1 |  | 1 | 0 | 0 | 1 | 3 | 10 | 3 | 20 | 23 | 0 |
| 4 | 1 | 21 | 1 | 400 | 290 | 0.37 | 2004/2/23 | 0 | 0 | 1 |  | 1 | 0 | 0 | 1 | 5 | 2 | 3 | 22 | 25 | 0 |
| 5 | 2 | 56 | 3 | 210 | 237 | 1 | 2004/4/14 | 1 | 0 | 2 |  | 0 | 0 | 0 | 7 | 9 | 9 | 9 | 0 | 9 | 1 |
| 6 | 1 | 24 | 3 | 420 | 63 | 0.12 | 2004/7/17 | 0 | 0 | 1 |  | 1 | 0 | 0 | 0 | 6 | 9 | 8 | 33 | 41 | 0 |
| 7 | 1 | 18 | 5 | 415 | 3 | 0 | 2004/11/5 | 0 | 0 | 1 |  | 1 | 0 | 0 | 0 | 5 | 2 | 2 | 12 | 14 | 1 |
| 8 | 2 | 19 | 13 | 190 | 6 | 0.23 | 2004/12/16 | 0 | 1 | 1 |  | 1 | 1 | 0 | 20 | 21 | 25 | 25 | 86 | 111 | 1 |
| 9 | 1 | 22 | 1 | 410 | 190 | 0.49 | 2005/2/21 | 0 | 0 | 1 |  | 1 | 0 | 0 | 10 | 18 | 8 | 6 | 35 | 41 | 0 |
| 10 | 2 | 66 | 9 | 110 | 8 | 0.45 | 2005/5/12 | 0 | 0 | 1 |  | 1 | 1 | 0 | 0 | 5 | 2 | 2 | 4 | 5 | 1 |
| 11 | 1 | 25 | 0 | 150 | 9 | 0.22 | 2005/6/13 | 1 | 1 | 1 |  | 1 | 0 | 0 | 22 | 29 | 22 | 22 | 122 | 144 | 1 |
| 12 | 1 | 25 | 2 | 70 | 3 | 0.51 | 2005/8/9 | 0 | 0 | 1 |  | 1 | 1 | 0 | 0 | 17 | 2 | 2 | 18 | 19 | 1 |
| 13 | 1 | 19 | 4 | 100 | 12 | 0.59 | 2005/8/10 | 1 | 1 | 1 |  | 1 | 0 | 0 | 6 | 111 | 111 | 111 | 0 | 111 | 1 |
| 14 | 2 | 21 | 0 | 90 | 28 | 0 | 2005/12/1 | 0 | 0 | 1 |  | 1 | 0 | 0 | 5 | 29 | 31 | 31 | 21 | 52 | 0 |
| 15 | 2 | 58 | 8 | 60 | 29 | 2 | 2006/7/5 | 0 | 0 | 4 |  | 0 | 1 | 0 | 5 | 3 | 3 | 3 | 0 | 3 | 1 |
| 16 | 2 | 55 | 3 | 140 | 7 | 0.19 | 2006/8/9 | 1 | 0 | 1 |  | 1 | 0 | 0 | 5 | 6 | 4 | 4 | 15 | 19 | 1 |
| 17 | 2 | 13 | 3 | 85 | 7 | 0 | 2006/8/20 | 0 | 0 | 4 |  | 0 | 1 | 0 | 5 | 2 | 2 | 2 | 0 | 2 | 1 |
| 18 | 1 | 35 | 1 | 200 | 0 | 0 | 2006/8/21 | 0 | 0 | 1 |  | 1 | 0 | 0 | 2 | 4 | 4 | 4 | 6 | 10 | 0 |
| 19 | 1 | 35 | 2 | 425 | 5 | 0.11 | 2006/12/4 | 1 | 0 | 1 |  | 1 | 0 | 0 | 1 | 2 | 2 | 2 | 0 | 1 | 1 |
| 20 | 2 | 22 | 3 | 210 | 230 | 0.46 | 2007/4/15 | 1 | 0 | 4 |  | 0 | 0 | 0 | 0 | 26 | 10 | 10 | 16 | 26 | 1 |
| 21 | 1 | 31 | 3 | 120 | 8 | 0.49 | 2007/5/3 | 0 | 0 | 1 |  | 1 | 1 | 0 | 0 | 2 | 2 | 2 | 11 | 13 | 0 |
| 22 | 1 | 22 | 14 | 90 | 7 | 0.18 | 2007/8/19 | 1 | 0 | 1 |  | 1 | 0 | 0 | 0 | 51 | 30 | 30 | 21 | 51 | 1 |
| 23 | 1 | 59 | 1 | 200 | 150 | 0.47 | 2007/8/24 | 0 | 0 | 1 |  | 1 | 1 | 0 | 0 | 5 | 7 | 7 | 21 | 28 | 0 |
| 24 | 1 | 22 | 9 | 270 | 110 | 1 | 2007/9/10 | 1 | 0 | 1 |  | 1 | 0 | 0 | 2 | 25 | 26 | 26 | 53 | 79 | 1 |
| 25 | 1 | 59 | 7 | 425 | 46 | 0 | 2007/10/3 | 0 | 0 | 3 |  | 0 | 1 | 0 | 0 | 5 | 2 | 2 | 3 | 5 | 1 |
| 26 | 1 | 44 | 1 | 80 | 3 | 0.21 | 2007/10/25 | 0 | 0 | 1 |  | 1 | 0 | 0 | 0 | 4 | 2 | 2 | 4 | 6 | 0 |
| 27 | 1 | 42 | 0 | 405 | 9 | 0.28 | 2007/11/10 | 1 | 0 | 1 |  | 1 | 1 | 0 | 0 | 3 | 3 | 3 | 6 | 9 | 1 |
| 28 | 1 | 57 | 5 | 120 | 325 | 2 | 2008/1/13 | 1 | 0 | 1 |  | 1 | 0 | 0 | 2 | 5 | 4 | 4 | 1 | 5 | 1 |
| 29 | 1 | 39 | 8 | 90 | 126 | 2 | 2008/1/31 | 0 | 0 | 2 |  | 0 | 0 | 0 | 0 | 5 | 3 | 3 | 2 | 5 | 0 |
| 30 | 1 | 56 | 1 | 290 | 320 | 0.67 | 2008/2/13 | 1 | 0 | 4 |  | 0 | 1 | 0 | 3 | 30 | 20 | 20 | 10 | 30 | 1 |
| 31 | 1 | 31 | 4 | 220 | 85 | 0.49 | 2008/3/14 | 1 | 0 | 1 |  | 1 | 1 | 0 | 0 | 46 | 7 | 7 | 54 | 61 | 0 |
| 32 | 1 | 19 | 2 | 160 | 111 | 0.55 | 2008/5/8 | 0 | 0 | 2 |  | 0 | 0 | 0 | 0 | 5 | 2 | 2 | 24 | 25 | 0 |
| 33 | 2 | 15 | 7 | 100 | 54 | 0 | 2008/7/18 | 0 | 0 | 1 |  | 1 | 0 | 0 | 0 | 4 | 2 | 3 | 7 | 10 | 0 |
| 34 | 1 | 28 | 14 | 450 | 230 | 0 | 2008/8/30 | 1 | 0 | 1 |  | 1 | 0 | 0 | 0 | 8 | 2 | 8 | 0 | 8 | 1 |
| 35 | 1 | 18 | 10 | 105 | 0 | 0.31 | 2008/9/7 | 0 | 0 | 1 |  | 1 | 0 | 0 | 0 | 4 | 2 | 2 | 3 | 4 | 1 |
| 36 | 2 | 35 | 3 | 443 | 180 | 0.49 | 2008/10/2 | 0 | 0 | 4 |  | 0 | 0 | 0 | 0 | 23 | 12 | 12 | 27 | 39 | 0 |
| 37 | 2 | 59 | 3 | 106 | 7 | 0.23 | 2008/10/17 | 1 | 1 | 1 |  | 1 | 1 | 0 | 0 | 4 | 4 | 4 | 29 | 33 | 0 |
| 38 | 1 | 43 | 8 | 250 | 48 | 0.57 | 2008/12/1 | 0 | 0 | 3 |  | 0 | 1 | 0 | 3 | 33 | 6 | 6 | 53 | 123 | 0 |
| 39 | 1 | 26 | 15 | 265 | 110 | 2 | 2008/12/29 | 1 | 0 | 4 |  | 0 | 0 | 0 | 3 | 2 | 2 | 2 | 1 | 2 | 1 |
| 40 | 1 | 48 | 0 | 140 | 6 | 6 | 2009/2/7 | 0 | 0 | 2 |  | 0 | 0 | 0 | 0 | 11 | 3 | 3 | 9 | 26 | 0 |
| 41 | 1 | 20 | 2 | 150 | 55 | 0.11 | 2009/3/20 | 0 | 0 | 1 |  | 1 | 1 | 0 | 0 | 21 | 2 | 3 | 25 | 28 | 1 |
| 42 | 1 | 26 | 12 | 130 | 33 | 0.71 | 2009/4/17 | 0 | 0 | 1 |  | 1 | 0 | 0 | 0 | 2 | 4 | 4 | 3 | 34 | 0 |
| 43 | 1 | 39 | 5 | 250 | 26 | 0 | 2009/5/3 | 0 | 1 | 1 |  | 1 | 1 | 0 | 0 | 8 | 8 | 8 | 0 | 8 | 1 |
| 44 | 1 | 53 | 7 | 100 | 6 | 0.17 | 2009/5/3 | 0 | 0 | 1 |  | 1 | 0 | 0 | 0 | 8 | 8 | 8 | 0 | 8 | 0 |
| 45 | 2 | 34 | 7 | 370 | 216 | 0.73 | 2009/5/11 | 1 | 0 | 3 |  | 0 | 1 | 0 | 0 | 82 | 5 | 5 | 104 | 141 | 0 |
| 46 | 1 | 26 | 15 | 230 | 410 | 1 | 2009/5/23 | 0 | 0 | 1 |  | 1 | 0 | 0 | 4 | 6 | 8 | 8 | 33 | 41 | 0 |
| 47 | 2 | 23 | 4 | 148 | 245 | 0.52 | 2009/8/8 | 0 | 0 | 2 |  | 0 | 0 | 0 | 0 | 5 | 5 | 5 | 5 | 33 | 0 |
| 48 | 1 | 19 | 3 | 110 | 80 | 1 | 2009/8/20 | 0 | 1 | 4 |  | 0 | 0 | 0 | 0 | 59 | 13 | 13 | 77 | 90 | 1 |
| 49 | 2 | 20 | 3 | 98 | 96 | 0.57 | 2009/8/30 | 1 | 0 | 1 |  | 1 | 0 | 0 | 0 | 11 | 9 | 9 | 15 | 24 | 0 |
| 50 | 1 | 61 | 10 | 417 | 6 | 0.14 | 2009/12/1 | 0 | 0 | 2 |  | 0 | 0 | 0 | 0 | 2 | 2 | 2 | 0 | 1 | 0 |
| 51 | 2 | 36 | 7 | 280 | 425 | 0.72 | 2010/5/11 | 0 | 0 | 2 |  | 0 | 0 | 0 | 0 | 6 | 6 | 6 | 17 | 23 | 0 |
| 52 | 1 | 23 | 3 | 408 | 21 | 0.49 | 2010/5/15 | 0 | 0 | 3 |  | 0 | 0 | 0 | 0 | 13 | 3 | 3 | 133 | 136 | 0 |
| 53 | 2 | 31 | 3 | 360 | 4 | 0 | 2010/6/24 | 1 | 0 | 1 |  | 1 | 1 | 0 | 3 | 16 | 16 | 16 | 5 | 34 | 1 |
| 54 | 1 | 53 | 4 | 240 | 25 | 0.61 | 2010/7/10 | 0 | 0 | 1 |  | 1 | 0 | 0 | 0 | 17 | 17 | 17 | 0 | 17 | 1 |
| 55 | 2 | 42 | 5 | 115 | 8 | 0.49 | 2010/7/29 | 1 | 0 | 1 |  | 1 | 0 | 0 | 0 | 1 | 16 | 16 | 13 | 29 | 0 |
| 56 | 2 | 69 | 12 | 432 | 9 | 0.18 | 2010/8/5 | 0 | 0 | 1 |  | 1 | 1 | 0 | 0 | 13 | 13 | 13 | 0 | 13 | 1 |
| 57 | 2 | 19 | 6 | 170 | 7 | 0.22 | 2010/8/12 | 0 | 0 | 1 |  | 1 | 0 | 0 | 0 | 11 | 4 | 4 | 10 | 14 | 0 |
| 58 | 1 | 32 | 2 | 335 | 355 | 0.63 | 2010/8/29 | 1 | 0 | 2 |  | 0 | 0 | 0 | 0 | 12 | 4 | 4 | 28 | 32 | 0 |
| 59 | 1 | 45 | 4 | 200 | 200 | 1 | 2010/10/5 | 0 | 0 | 1 |  | 1 | 0 | 0 | 0 | 5 | 4 | 4 | 28 | 35 | 0 |
| 60 | 1 | 18 | 6 | 180 | 13 | 0.17 | 2010/10/8 | 1 | 0 | 1 |  | 1 | 0 | 0 | 0 | 3 | 4 | 4 | 0 | 4 | 0 |
| 61 | 2 | 20 | 1 | 320 | 8 | 0.21 | 2010/10/23 | 0 | 0 | 1 |  | 1 | 1 | 0 | 0 | 10 | 5 | 5 | 15 | 20 | 0 |
| 62 | 1 | 21 | 20 | 300 | 0 | 0.51 | 2010/11/15 | 1 | 1 | 1 |  | 1 | 0 | 0 | 0 | 41 | 14 | 14 | 27 | 41 | 1 |
| 63 | 1 | 23 | 4 | 125 | 3 | 0 | 2010/11/18 | 1 | 0 | 1 |  | 1 | 1 | 0 | 0 | 6 | 2 | 2 | 6 | 7 | 0 |
| 64 | 1 | 58 | 7 | 405 | 4 | 0.74 | 2010/11/26 | 1 | 0 | 1 |  | 1 | 1 | 0 | 0 | 3 | 3 | 3 | 25 | 28 | 0 |
| 65 | 2 | 18 | 8 | 400 | 410 | 1 | 2010/12/9 | 0 | 0 | 1 |  | 1 | 0 | 0 | 0 | 20 | 6 | 6 | 31 | 38 | 0 |
| 66 | 1 | 36 | 5 | 98 | 9 | 0.53 | 2011/1/11 | 0 | 0 | 1 |  | 1 | 1 | 0 | 0 | 1 | 2 | 2 | 0 | 14 | 0 |
| 67 | 2 | 58 | 10 | 360 | 360 | 4 | 2011/1/17 | 0 | 0 | 4 |  | 0 | 0 | 0 | 0 | 59 | 5 | 5 | 54 | 59 | 1 |
| 68 | 1 | 27 | 3 | 170 | 8 | 1 | 2011/2/12 | 1 | 0 | 1 |  | 1 | 1 | 0 | 0 | 37 | 2 | 9 | 48 | 58 | 0 |
| 69 | 1 | 29 | 6 | 408 | 89 | 0.46 | 2011/5/6 | 0 | 1 | 4 |  | 0 | 0 | 0 | 0 | 45 | 20 | 20 | 25 | 45 | 1 |
| 70 | 1 | 16 | 1 | 400 | 30 | 0.83 | 2011/5/10 | 0 | 0 | 1 |  | 1 | 1 | 0 | 0 | 7 | 6 | 6 | 8 | 14 | 0 |
| 71 | 1 | 20 | 4 | 155 | 14 |  | 2011/7/26 | 0 | 0 | 1 |  | 1 | 0 | 0 | 0 | 10 | 2 | 3 | 14 | 17 | 0 |
| 72 | 1 | 65 | 2 | 200 | 50 | 4 | 2011/7/29 | 1 | 0 | 1 |  | 1 | 1 | 0 | 0 | 19 | 2 | 19 | 0 | 19 | 1 |
| 73 | 1 | 48 | 5 | 90 | 19 | 0.55 | 2011/8/14 | 0 | 0 | 1 |  | 1 | 0 | 0 | 0 | 10 | 3 | 3 | 20 | 23 | 1 |
| 74 | 1 | 23 | 1 | 100 | 29 | 0.71 | 2011/9/5 | 0 | 0 | 4 |  | 0 | 1 | 0 | 0 | 10 | 2 | 2 | 9 | 10 | 1 |
| 75 | 2 | 23 | 3 | 300 | 90 | 0.46 | 2011/12/22 | 0 | 0 | 1 |  | 1 | 0 | 0 | 7 | 95 | 2 | 48 | 142 | 190 | 1 |
| 76 | 1 | 30 | 6 | 380 | 182 | 1 | 2012/2/5 | 0 | 0 | 4 |  | 0 | 1 | 0 | 1 | 18 | 17 | 17 | 15 | 32 | 0 |
| 77 | 1 | 51 | 3 | 155 | 13 | 0.47 | 2012/3/1 | 0 | 1 | 1 |  | 1 | 0 | 0 | 2 | 12 | 7 | 7 | 25 | 32 | 0 |
| 78 | 1 | 26 | 3 | 118 | 3 | 0.31 | 2012/3/9 | 0 | 0 | 1 |  | 1 | 1 | 0 | 0 | 25 | 8 | 8 | 23 | 32 | 0 |
| 79 | 1 | 13 | 1 | 145 | 58 | 0 | 2012/3/15 | 0 | 0 | 1 |  | 1 | 0 | 0 | 0 | 24 | 4 | 4 | 21 | 25 | 0 |
| 80 | 2 | 81 | 5 | 135 | 11 | 0.51 | 2012/3/28 | 1 | 0 | 1 |  | 1 | 1 | 0 | 0 | 31 | 5 | 5 | 48 | 53 | 1 |
| 81 | 1 | 19 | 3 | 170 | 54 | 0.14 | 2012/4/18 | 1 | 0 | 1 |  | 1 | 0 | 0 | 1 | 5 | 5 | 5 | 19 | 24 | 0 |
| 82 | 1 | 51 | 1 | 110 | 248 | 5 | 2012/4/27 | 0 | 0 | 2 |  | 0 | 1 | 0 | 0 | 39 | 9 | 9 | 30 | 39 | 1 |
| 83 | 2 | 40 | 6 | 409 | 8 | 0.23 | 2012/5/23 | 0 | 1 | 1 |  | 1 | 0 | 0 | 0 | 3 | 3 | 3 | 0 | 3 | 0 |
| 84 | 1 | 18 | 5 | 200 | 9 | 0 | 2012/5/27 | 0 | 0 | 2 |  | 0 | 1 | 0 | 0 | 46 | 15 | 15 | 78 | 93 | 0 |
| 85 | 1 | 50 | 3 | 250 | 150 | 1 | 2012/6/11 | 0 | 0 | 1 |  | 1 | 0 | 0 | 0 | 42 | 19 | 19 | 30 | 49 | 0 |
| 86 | 1 | 40 | 1 | 180 | 4 | 0.12 | 2012/6/18 | 0 | 0 | 1 |  | 1 | 1 | 0 | 0 | 16 | 7 | 7 | 12 | 19 | 1 |
| 87 | 1 | 20 | 4 | 145 | 235 | 0.48 | 2012/7/15 | 0 | 0 | 1 |  | 1 | 0 | 0 | 1 | 23 | 2 | 14 | 11 | 25 | 0 |
| 88 | 1 | 29 | 1 | 410 | 243 | 2 | 2012/9/27 | 0 | 0 | 2 |  | 0 | 1 | 0 | 0 | 16 | 3 | 3 | 14 | 17 | 0 |
| 89 | 1 | 33 | 2 | 270 | 3 | 0.73 | 2013/1/26 | 1 | 0 | 1 |  | 1 | 0 | 0 | 0 | 8 | 2 | 4 | 7 | 11 | 0 |
| 90 | 2 | 20 | 1 | 120 | 365 | 3 | 2013/2/4 | 0 | 0 | 2 |  | 0 | 0 | 0 | 0 | 13 | 6 | 6 | 9 | 15 | 0 |
| 91 | 1 | 48 | 6 | 100 | 4 | 0 | 2013/3/17 | 1 | 1 | 1 |  | 1 | 1 | 0 | 1 | 14 | 2 | 2 | 57 | 58 | 0 |
| 92 | 1 | 54 | 5 | 175 | 5 | 0.17 | 2013/4/19 | 0 | 0 | 1 |  | 1 | 0 | 0 | 0 | 5 | 2 | 2 | 6 | 8 | 1 |
| 93 | 2 | 39 | 1 | 140 | 3 | 0 | 2013/4/27 | 0 | 0 | 1 |  | 1 | 1 | 0 | 0 | 18 | 2 | 2 | 16 | 18 | 1 |
| 94 | 1 | 36 | 15 | 125 | 1 | 0.62 | 2013/5/15 | 0 | 0 | 1 |  | 1 | 0 | 0 | 0 | 9 | 6 | 6 | 19 | 25 | 0 |
| 95 | 2 | 21 | 15 | 250 | 9 | 0.12 | 2013/6/21 | 0 | 0 | 1 |  | 1 | 1 | 0 | 0 | 14 | 2 | 4 | 22 | 26 | 0 |
| 96 | 1 | 23 | 1 | 170 | 19 | 0.73 | 2013/6/28 | 1 | 0 | 1 |  | 1 | 0 | 0 | 4 | 28 | 23 | 23 | 7 | 30 | 1 |
| 97 | 1 | 22 | 4 | 190 | 169 | 0.48 | 2013/7/10 | 0 | 0 | 1 |  | 1 | 1 | 0 | 3 | 137 | 103 | 105 | 32 | 137 | 1 |
| 98 | 1 | 67 | 1 | 422 | 320 | 1 | 2013/7/16 | 0 | 0 | 1 |  | 1 | 0 | 0 | 2 | 83 | 56 | 56 | 41 | 97 | 1 |
| 99 | 2 | 33 | 10 | 140 | 150 | 0.47 | 2013/7/23 | 0 | 0 | 1 |  | 1 | 0 | 0 | 0 | 4 | 4 | 4 | 16 | 20 | 0 |
| 100 | 2 | 31 | 5 | 280 | 208 | 0.79 | 2013/7/24 | 1 | 0 | 1 |  | 1 | 1 | 0 | 0 | 7 | 3 | 3 | 0 | 4 | 0 |
| 101 | 1 | 53 | 10 | 190 | 74 | 0.61 | 2013/10/11 | 0 | 0 | 2 |  | 0 | 0 | 0 | 0 | 7 | 7 | 8 | 21 | 29 | 0 |
| 102 | 2 | 50 | 15 | 417 | 75 | 0.57 | 2013/10/12 | 1 | 0 | 3 |  | 0 | 0 | 0 | 0 | 91 | 9 | 9 | 91 | 100 | 1 |
| 103 | 1 | 22 | 10 | 409 | 240 | 1 | 2013/12/20 | 0 | 0 | 4 |  | 0 | 1 | 0 | 0 | 27 | 5 | 5 | 34 | 39 | 1 |
| 104 | 1 | 27 | 1 | 265 | 465 | 1 | 2014/1/26 | 1 | 1 | 2 |  | 0 | 0 | 0 | 1 | 16 | 3 | 3 | 55 | 58 | 0 |
| 105 | 1 | 43 | 1 | 370 | 145 | 10 | 2014/2/2 | 0 | 0 | 2 |  | 0 | 1 | 0 | 0 | 13 | 12 | 10 | 12 | 22 | 1 |
| 106 | 1 | 18 | 2 | 412 | 7 | 1 | 2014/2/20 | 1 | 0 | 2 |  | 0 | 0 | 0 | 0 | 13 | 2 | 5 | 49 | 54 | 0 |
| 107 | 1 | 31 | 4 | 390 | 168 | 0.62 | 2014/2/26 | 0 | 0 | 2 |  | 0 | 0 | 0 | 0 | 8 | 2 | 2 | 15 | 17 | 0 |
| 108 | 2 | 23 | 2 | 160 | 2 | 0.15 | 2014/3/16 | 0 | 0 | 1 |  | 1 | 0 | 0 | 0 | 11 | 4 | 4 | 16 | 20 | 0 |
| 109 | 1 | 23 | 3 | 295 | 203 | 0.55 | 2014/4/16 | 0 | 1 | 2 |  | 0 | 1 | 0 | 0 | 17 | 7 | 7 | 11 | 18 | 0 |
| 110 | 1 | 24 | 3 | 432 | 7 | 0.22 | 2004/10/7 | 1 | 0 | 1 |  | 1 | 1 | 1 | 0 | 2 | 2 | 2 | 0 | 1 | 1 |
| 111 | 1 | 52 | 4 | 128 | 11 | 0.49 | 2005/7/11 | 0 | 1 | 1 |  | 1 | 1 | 1 | 32 | 22 | 22 | 22 | 0 | 22 | 0 |
| 112 | 2 | 21 | 15 | 400 | 3 | 0.12 | 2006/9/4 | 1 | 0 | 1 |  | 1 | 1 | 1 | 5 | 6 | 6 | 6 | 0 | 6 | 0 |
| 113 | 1 | 52 | 2 | 406 | 8 | 0.14 | 2007/1/2 | 1 | 0 | 1 |  | 1 | 1 | 1 | 0 | 2 | 2 | 2 | 0 | 1 | 0 |
| 114 | 1 | 17 | 3 | 210 | 690 | 2 | 2007/2/17 | 1 | 1 | 2 |  | 0 | 1 | 1 | 3 | 2 | 2 | 2 | 1 | 2 | 0 |
| 115 | 1 | 27 | 5 | 413 | 480 | 0 | 2008/1/10 | 1 | 0 | 2 |  | 0 | 1 | 1 | 1 | 3 | 3 | 3 | 0 | 3 | 0 |
| 116 | 1 | 30 | 7 | 310 | 73 | 0.48 | 2008/12/9 | 0 | 1 | 1 |  | 1 | 1 | 1 | 1 | 9 | 9 | 9 | 1 | 11 | 0 |
| 117 | 1 | 48 | 6 | 180 | 598 | 0.77 | 2008/12/22 | 1 | 1 | 2 |  | 0 | 1 | 1 | 0 | 27 | 14 | 14 | 13 | 27 | 0 |
| 118 | 2 | 19 | 9 | 290 | 128 | 3 | 2009/3/8 | 0 | 0 | 1 |  | 1 | 1 | 1 | 4 | 4 | 4 | 4 | 0 | 4 | 0 |
| 119 | 2 | 18 | 18 | 220 | 4 | 0 | 2009/9/9 | 1 | 1 | 1 |  | 1 | 1 | 1 | 6 | 25 | 22 | 22 | 5 | 27 | 1 |
| 120 | 2 | 20 | 4 | 412 | 8 | 0.34 | 2009/9/29 | 1 | 0 | 4 |  | 0 | 1 | 1 | 3 | 2 | 2 | 2 | 0 | 1 | 1 |
| 121 | 1 | 74 | 3 | 185 | 2 | 0 | 2011/1/23 | 0 | 1 | 1 |  | 1 | 1 | 1 | 0 | 45 | 44 | 44 | 1 | 45 | 0 |
| 122 | 1 | 37 | 1 | 404 | 8 | 0 | 2012/2/27 | 1 | 0 | 2 |  | 0 | 1 | 1 | 1 | 13 | 14 | 14 | 0 | 14 | 1 |
| 123 | 1 | 54 | 10 | 130 | 104 | 1.97 | 2022/4/22 | 0 | 1 | 1 | EBV | 1 | 1 | 0 | 0 | 0 | 11 | 11 | 0 | 11 | 0 |
| 124 | 1 | 56 | 4 | 230 | 41 | 1.82 | 2022/4/7 | 1 | 1 | 3 | Cryptococcus neoformans | 0 | 1 | 0 | 0 | 38 | 56 | 56 | 0 | 56 | 1 |
| 125 | 1 | 51 | 1 | 270 | 57 | 0.79 | 2020/1/12 | 0 | 1 | 1 | EBV | 1 | 1 | 0 | 0 | 0 | 9 | 5 | 4 | 9 | 0 |
| 126 | 1 | 16 | 3 | 211 | 131 | 0.48 | 2017/9/11 | 1 | 1 | 1 |  | 1 | 1 | 0 | 0 | 3 | 3 | 3 | 7 | 16 | 0 |
| 127 | 2 | 39 | 1 | 220 | 3 | 0 | 2017/06/26 | 1 | 1 | 1 |  | 1 | 1 | 0 | 0 | 0 | 18 | 3 | 16 | 30 | 0 |
| 128 | 1 | 68 | 7 | 300 | 235 | 0.66 | 2020/10/5 | 0 | 0 | 1 | EBV | 1 | 0 | 0 | 0 | 0 | 10 | 0 | 10 | 10 | 0 |
| 129 | 1 | 66 | 5 | 120 | 85 | 1.84 | 2022/3/19 | 0 | 1 | 1 | Herpes simplex virus | 1 | 1 | 0 | 0 | 0 | 4 | 4 | 12 | 16 | 0 |
| 130 | 2 | 39 | 4 | 370 | 196 | 0.56 | 2020/9/11 | 1 | 1 | 1 | Cytomegalovirus | 1 | 1 | 0 | 3 | 1 | 6 | 7 | 0 | 7 | 1 |
| 131 | 1 | 25 | 1 | 270 | 12 | 0.17 | 2019/12/20 | 0 | 1 | 1 |  | 1 | 1 | 1 | 1 | 16 | 17 | 16 | 1 | 17 | 1 |
| 132 | 2 | 23 | 4 | 180 | 284 | 1.28 | 2018/01/10 | 0 | ND | 1 |  | 1 | 0 | 0 | 0 | 0 | 11 | 4 | 7 | 11 | 0 |
| 133 | 1 | 51 | 4 | 160 | 0 | 0 | 2017/08/16 | 0 | ND | 1 |  | 1 | 0 | 0 | 0 | 0 | 11 | 2 | 2 | 11 | 0 |
| 134 | 2 | 79 | 3 | 140 | 0 | 0.38 | 2017/1/10 | 0 | 0 | 1 |  | 1 | 1 | 0 | 0 | 0 | 9 | 9 | 0 | 9 | 0 |
| 135 | 1 | 23 | 1 | 177 | 36 | 0.51 | 2017/02/26 | ND | ND | 1 |  | 1 | 1 | 0 | 0 | 0 | 8 | 4 | 4 | 8 | 0 |
| 136 | 1 | 19 | 2 | 160 | 50 | 0.71 | 2016/11/03 | 0 | 1 | 1 |  | 1 | 1 | 0 | 0 | 5 | 11 | 11 | 10 | 21 | 0 |
| 137 | 2 | 20 | 4 | 160 | 16 | 0.35 | 2018/12/07 | 0 | 1 | 1 |  | 1 | 0 | 0 | 0 | 0 | 12 | 12 | 0 | 12 | 0 |
| 138 | 1 | 34 | 7 | 340 | 78 | 1.76 | 2020/10/31 | 0 | 0 | 1 | EBV | 1 | 1 | 0 | 0 | 0 | 18 | 0 | 18 | 18 | 0 |
| 139 | 1 | 47 | 0.5 | 140 | 25 | 0.84 | 2019/04/18 | 1 | 1 | 1 |  | 1 | 1 | 0 | 0 | 0 | 18 | 11 | 7 | 18 | 0 |
| 140 | 1 | 80 | 5 | 104 | 3 | 0.76 | 2021/05/01 | 1 | 1 | 1 |  | 1 | 1 | 0 | 0 | 0 | 6 | 0 | 6 | 6 | 0 |
| 141 | 1 | 54 | 7 | 220 | 21 | 0.82 | 2018/08/27 | 0 | 0 | 1 |  | 1 | 1 | 0 | 2 | 11 | 42 | 42 | 0 | 42 | 1 |
| 142 | 2 | 72 | 8 | 160 | 165 | 1.65 | 2021/08/19 | 1 | 1 | 1 | EBV | 1 | 1 | 0 | 0 | 0 | 39 | 8 | 21 | 29 | 0 |
| 143 | 1 | 72 | 3 | 90 | 44 | 0.65 | 2015/08/28 | 0 | 0 | 1 |  | 1 | 0 | 0 | 1 | 0 | 10 | 7 | 3 | 10 | 0 |
| 144 | 2 | 22 | 3 | 180 | 1 | 0.35 | 2021/05/29 | 0 | 1 | 1 |  | 1 | 0 | 0 | 0 | 4 | 12 | 4 | 8 | 12 | 0 |
| 145 | 1 | 35 | 4 | 100 | 5 | 1.28 | 2018/02/27 | 1 | 1 | 1 |  | 1 | 1 | 0 | 1 | 31 | 56 | 39 | 17 | 56 | 1 |
| 146 | 2 | 51 | 4 | 160 | 18 | 0.96 | 2018/07/09 | 0 | 1 | 1 |  | 1 | 1 | 0 | 0 | 0 | 11 | 11 | 0 | 11 | 1 |
| 147 | 2 | 48 | 1 | 160 | 43 | 0.44 | 2017/08/17 | 0 | ND | 1 |  | 1 | 0 | 0 | 1 | 0 | 12 | 12 | 0 | 12 | 0 |
| 148 | 2 | 64 | 1 | 260 | 200 | 1.44 | 2021/01/02 | 1 | 1 | 2 | Streptococcus pneumoniae | 0 | 1 | 0 | 0 | 2 | 6 | 3 | 11 | 25 | 1 |
| 149 | 1 | 52 | 1 | 40 | 41 | 1.99 | 2018/08/27 | 1 | 1 | 2 |  | 0 | 1 | 0 | 3 | 7 | 68 | 60 | 8 | 68 | 1 |
| 150 | 2 | 45 | 0.5 | 158 | 5209 | 2.36 | 2020/12/06 | 1 | 0 | 2 | Neisseria meningitidis | 0 | 1 | 0 | 0 | 0 | 9 | 9 | 0 | 9 | 0 |
| 151 | 2 | 53 | 0.41 | 140 | 5792 | 3.84 | 2019/11/01 | 1 | 1 | 2 |  | 0 | 1 | 0 | 0 | 4 | 18 | 4 | 14 | 18 | 0 |
| 152 | 1 | 31 | 2 | 220 | 1052 | 1.04 | 2021/7/3 | 1 | 0 | 2 | Listeria monocytogenes | 0 | 1 | 0 | 0 | 0 | 38 | 20 | 18 | 38 | 0 |
| 153 | 2 | 53 | 1 | 239 | 1 | 0.28 | 2021/11/24 | 0 | 0 | 1 | Cytomegalovirus | 1 | 1 | 0 | 0 | 0 | 6 | 6 | 9 | 15 | 0 |
| 154 | 1 | 55 | 4 | 270 | 152 | 1.04 | 2018/06/07 | 1 | 0 | 1 |  | 1 | 1 | 0 | 0 | 0 | 11 | 11 | 16 | 26 | 0 |
| 155 | 2 | 14 | 4 | 300 | 26 | 0.33 | 2020/06/16 | 1 | 1 | 1 | Herpes simplex virus | 1 | 1 | 0 | 2 | 18 | 31 | 31 | 0 | 31 | 0 |
| 156 | 1 | 63 | 15 | 200 | 41 | 1.42 | 2019/12/15 | 1 | 1 | 1 | Herpes simplex virus | 1 | 1 | 1 | 2 | 15 | 39 | 39 | 0 | 39 | 1 |
| 157 | 2 | 56 | 7 | 200 | 5 | 0.62 | 2019/05/26 | ND | 1 | 1 |  | 1 | 1 | 1 | 3 | 4 | 4 | 4 | 0 | 4 | 1 |
| 158 | 1 | 55 | 1 | 354 | 368 | 2.53 | 2019/12/12 | 1 | 1 | 1 | varicella-zoster virus | 1 | 1 | 1 | 1 | 6 | 7 | 7 | 0 | 7 | 1 |
| 159 | 1 | 30 | 30 | 219 | 21 | 0.34 | 2014/09/15 | ND | 1 | 1 | epidemic type B encephalitis virus | 1 | 1 | 0 | 2 | 0 | 174 | 174 | 0 | 174 | 1 |
| 160 | 2 | 54 | 1 | 140 | 424 | 0.99 | 2019/03/10 | 1 | 1 | 2 |  | 0 | 1 | 0 | 0 | 0 | 14 | 2 | 12 | 14 | 0 |
| 161 | 2 | 45 | 4 | 280 |  | 3.72 | 2014/02/22 | ND | ND | 2 |  | 0 | 1 | 1 | 1 | 2 | 2 | 2 | 0 | 2 | 1 |
| 162 | 1 | 38 | 3 | 350 | 43 | 0.46 | 2021/12/27 | 1 | 1 | 1 |  | 1 | 1 | 0 | 0 | 0 | 7 | 7 | 2 | 9 | 0 |
| 163 | 1 | 19 | 7 | 140 | 396 | 1.08 | 2021/11/26 | 1 | 0 | 1 |  | 1 | 0 | 0 | 0 | 0 | 2 | 2 | 14 | 16 | 0 |
| 164 | 1 | 64 | 2 | 120 | 125 | 1.93 | 2021/12/2 | 0 | 0 | 1 | EBV | 1 | 1 | 0 | 0 | 0 | 7 | 7 | 0 | 7 | 0 |
| 165 | 2 | 40 | 3 | 240 | 168 | 0.44 | 2021/12/11 | 1 | 1 | 1 | Herpes simplex virus | 1 | 1 | 0 | 0 | 0 | 3 | 3 | 6 | 9 | 1 |
| 166 | 2 | 46 | 2 | 530 | 1046 | 5.06 | 2021/8/24 | 0 | 0 | 2 |  | 0 | 1 | 0 | 0 | 10 | 13 | 13 | 2 | 15 | 1 |
| 167 | 1 | 57 | 2 | 200 | 1700 | 1.86 | 2021/7/16 | 0 | 0 | 2 | klebsiella | 0 | 0 | 0 | 0 | 12 | 12 | 12 | 10 | 22 | 0 |
| 168 | 1 | 41 | 3 | 266 | 284 | 0.6 | 2021/8/18 | 0 | 1 | 1 |  | 1 | 1 | 0 | 0 | 12 | 12 | 12 | 7 | 19 | 0 |
| 169 | 1 | 22 | 2 | 140 | 1 | 0.29 | 2021/6/7 | 0 | 0 | 1 | Herpes simplex virus | 1 | 0 | 0 | 0 | 0 | 2 | 2 | 6 | 8 | 0 |
| 170 | 2 | 33 | 5 | 160 | 5 | 0.54 | 2021/6/21 | 0 | 0 | 1 |  | 1 | 0 | 0 | 0 | 0 | 4 | 4 | 5 | 9 | 0 |
| 171 | 1 | 32 | 7 | 140 | 4 | 0.23 | 2021/7/22 | 0 | 1 | 1 |  | 1 | 1 | 0 | 0 | 3 | 8 | 8 | 0 | 8 | 0 |
| 172 | 1 | 59 | 1 | 200 | 31 | 1.07 | 2021/6/25 | 0 | 1 | 1 |  | 1 | 0 | 0 | 0 | 0 | 8 | 8 | 3 | 11 | 0 |
| 173 | 1 | 15 | 11 | 105 | 1 | 0.27 | 2021/1/20 | 0 | 0 | 1 |  | 1 | 1 | 0 | 0 | 0 | 13 | 13 | 0 | 13 | 0 |
| 174 | 1 | 68 | 0.29 | 130 | 94 | 2.21 | 2021/2/18 | 0 | 1 | 3 |  | 0 | 1 | 1 | 2 | 9 | 14 | 14 | 0 | 14 | 1 |
| 175 | 1 | 38 | 15 | 226 | 66 | 0.79 | 2020/9/1 | 1 | 1 | 3 | Cryptococcus neoformans | 0 | 1 | 1 | 0 | 2 | 14 | 14 | 0 | 14 | 1 |
| 176 | 1 | 69 | 14 | 170 | 231 | 1.96 | 2020/7/29 | 0 | 0 | 1 |  | 1 | 1 | 0 | 1 | 8 | 27 | 27 | 0 | 27 | 0 |
| 177 | 1 | 15 | 7 | 259 | 158 | 0.62 | 2020/6/21 | 0 | 1 | 1 | EBV | 1 | 1 | 0 | 0 | 0 | 8 | 8 | 11 | 19 | 0 |
| 178 | 2 | 19 | 7 | 160 | 30 | 0.71 | 2020/7/19 | 1 | 1 | 1 | Herpes simplex virus | 1 | 1 | 0 | 0 | 0 | 15 | 15 | 9 | 24 | 0 |
| 179 | 1 | 62 | 30 | 160 | 23 | 1.58 | 2020/5/22 | 1 | 0 | 4 | Mycobacterium tuberculosis | 0 | 1 | 0 | 0 | 0 | 4 | 3 | 1 | 4 | 1 |
| 180 | 1 | 50 | 6 |  | 8755 | 1.61 | 2020/7/20 | 0 | 1 | 2 | Streptococcus pneumoniae | 0 | 1 | 0 | 0 | 5 | 12 | 12 | 0 | 12 | 1 |
| 181 | 1 | 54 | 0.5 | 300 | 95 | 1.67 | 2020/1/17 | 1 | ND | 3 | Cryptococcus neoformans | 0 | 1 | 1 | 2 | 3 | 5 | 5 | 0 | 5 | 1 |
| 182 | 1 | 46 | 0.5 | 250 | 1936 | 2.37 | 2019/11/30 | 0 | 1 | 2 | klebsiella | 0 | 1 | 0 | 0 | 0 | 16 | 16 | 0 | 16 | 0 |
| 183 | 2 | 75 | 10 | 120 | 1 | 0.25 | 2019/10/19 | 0 | ND | 1 |  | 1 | 1 | 0 | 1 | 3 | 6 | 6 | 2 | 8 | 1 |
| 184 | 1 | 68 | 4 | 143 | 85 | 0.4 | 2019/7/8 | 0 | 0 | 1 |  | 1 | 0 | 0 | 0 | 0 | 2 | 2 | 9 | 11 | 0 |
| 185 | 1 | 59 | 1 | 120 | 23 | 0.46 | 2019/6/27 | 0 | 1 | 1 |  | 1 | 0 | 0 | 0 | 0 | 4 | 4 | 3 | 7 | 0 |
| 186 | 1 | 55 | 9 | 195 | 9903 | 2.82 | 2019/6/6 | 1 | 0 | 2 |  | 0 | 0 | 0 | 0 | 0 | 13 | 13 | 30 | 46 | 0 |
| 187 | 2 | 70 | 21 | 160 | 149 | 1.19 | 2019/3/26 | ND | ND | 3 | Cryptococcus neoformans | 0 | 1 | 0 | 0 | 0 | 2 | 2 | 0 | 2 | 1 |
| 188 | 1 | 41 | 10 | 166 | 150 | 0.48 | 2018/12/24 | 0 | 0 | 1 | Herpes simplex virus | 1 | 1 | 0 | 0 | 3 | 3 | 3 | 19 | 22 | 0 |
| 189 | 1 | 44 | 6 | 120 | 47 | 0.89 | 2019/2/9 | 0 | 1 | 1 |  | 1 | 1 | 0 | 0 | 0 | 16 | 6 | 11 | 17 | 0 |
| 190 | 1 | 52 | 3 | 182 | 188 | 3.37 | 2018/10/31 | 1 | 0 | 2 | Streptococcus pneumoniae | 0 | 1 | 0 | 0 | 0 | 23 | 23 | 2 | 25 | 1 |
| 191 | 1 | 44 | 0.33 | 180 | 8 | 0.71 | 2018/11/10 | 1 | 1 | 1 |  | 1 | 1 | 0 | 1 | 7 | 31 | 31 | 0 | 31 | 0 |
| 192 | 1 | 25 | 1 | 98 | 3 | 0.24 | 2018/11/7 | 0 | 1 | 1 |  | 1 | 1 | 0 | 0 | 0 | 14 | 14 | 0 | 14 | 0 |
| 193 | 1 | 40 | 3 | 180 | 6 | 1.64 | 2018/2/17 | ND | 1 | 1 |  | 1 | 0 | 0 | 0 | 4 | 4 | 4 | 0 | 4 | 0 |
| 194 | 2 | 29 | 4 | 90 | 22 | 0.22 | 2018/2/23 | 0 | 1 | 1 |  | 1 | 1 | 0 | 0 | 0 | 6 | 6 | 8 | 14 | 1 |
| 195 | 2 | 50 | 1 | 128 | 5 | 0.07 | 2018/2/19 | 0 | 0 | 1 |  | 1 | 0 | 0 | 0 | 0 | 3 | 3 | 4 | 10 | 0 |
| 196 | 1 | 41 | 3 | 240 | 83 | 2.61 | 2018/7/1 | 0 | 0 | 1 | Herpes simplex virus | 1 | 0 | 0 | 0 | 0 | 10 | 10 | 4 | 14 | 0 |
| 197 | 1 | 26 | 6 | 223 | 312 | 3 | 2018/1/2 | 1 | 0 | 4 | Mycobacterium tuberculosis | 0 | 0 | 0 | 0 | 0 | 7 | 7 | 0 | 7 | 0 |
| 198 | 2 | 65 | 3 | 204 | 2680 | 2.12 | 2018/1/10 | ND | ND | 2 |  | 0 | 1 | 0 | 0 | 4 | 4 | 4 | 0 | 4 | 1 |
| 199 | 1 | 54 | 32 | 199 | 172 | 0.64 | 2017/8/16 | 0 | 0 | 1 |  | 1 | 0 | 0 | 0 | 0 | 7 | 6 | 18 | 29 | 0 |
| 200 | 2 | 15 | 7 | 180 | 33 | 0.26 | 2016/5/2 | 0 | 1 | 1 |  | 1 | 1 | 0 | 0 | 14 | 16 | 16 | 3 | 21 | 0 |
| 201 | 2 | 48 | 3 | 175 | 76 | 0.1 | 2015/6/27 | 0 | 0 | 1 |  | 1 | 0 | 0 | 0 | 0 | 3 | 3 | 10 | 13 | 0 |
| 202 | 2 | 28 | 6 | 139 | 46 | 0.67 | 2015/6/18 | 0 | 0 | 1 |  | 1 | 0 | 0 | 0 | 0 | 3 | 3 | 4 | 13 | 0 |
| 203 | 2 | 33 | 7 | 120 | 7 | 0.1 | 2015/8/31 | 0 | 1 | 1 |  | 1 | 1 | 0 | 0 | 0 | 2 | 2 | 0 | 15 | 0 |
| 204 | 2 | 37 | 5 | 449 | 132 | 0.57 | 2015/8/30 | 0 | ND | 1 |  | 1 | 1 | 0 | 0 | 0 | 2 | 2 | 17 | 20 | 0 |
| 205 | 2 | 60 | 7 | 129 | 3 | 0.26 | 2015/4/28 | 0 | 0 | 1 |  | 1 | 0 | 0 | 0 | 0 | 2 | 2 | 0 | 5 | 0 |
| 206 | 2 | 57 | 4 | 180 | 77 | 1.16 | 2015/2/2 | 1 | ND | 1 |  | 1 | 0 | 0 | 0 | 0 | 5 | 5 | 9 | 14 | 1 |
| 207 | 2 | 24 | 2 | 160 | 12 | 0.35 | 2022/1/19 | 1 | 1 | 1 |  | 1 | 1 | 0 | 0 | 0 | 19 | 19 | 7 | 26 | 0 |
| 208 | 2 | 15 | 1 | 270 | 8 | 0.81 | 2022/1/27 | 1 | 1 | 1 | Human Herpes Virus 6 | 1 | 1 | 0 | 0 | 11 | 11 | 11 | 3 | 14 | 0 |
| 209 | 2 | 34 | 4 | 250 | 56 | 1.04 | 2021/12/10 | 1 | 1 | 4 | Mycobacterium tuberculosis | 0 | 1 | 1 | 3 | 3 | 13 | 8 | 0 | 13 | 1 |

Information on 209 patients with severe infectious encephalitis, including age, sex, CSF pressure, result of imaging scan, result of EEG, causative agent, etc
