# Supplementary material for: Predictors of mortality and poor outcome for patients with severe infectious encephalitis in the intensive care unit: a cross-sectional study
Source: BMC Infect Dis. 2024 Apr 22;24:421. doi: 10.1186/s12879-024-09312-1 (PMC11034050; doi:10.1186/s12879-024-09312-1)
Supplement: Supplementary file 2 — Supplementary Material 2 [file 12879_2024_9312_MOESM2_ESM.docx]

Table 5.Univariate analysis of mortality risk prediction for severe infectious encephalitis in the ICU

| Variables | OR (95% CI) | *P* |
| --- | --- | --- |
| Mean age (years) | 1.01(0.98-1.03) | 0.720 |
| Gender | 1.20(0.47-3.08) | 0.710 |
| Onset time (days) | 1.02(0.95-1.11) | 0.552 |
| Hospitalized days | 0.95(0.90-0.99) | 0.014 |
| Admission to ICU (days) | 1.00(0.98-1.03) | 0.968 |
| Days of critical condition | 0.97(0.93-1.01) | 0.142 |
| Number of rescuing | 1.13(1.02-1.24) | 0.017 |
| Days of advanced care | 1.00(0.98-1.03) | 0.922 |
| Days of primary care | 0.76(0.59-0.97) | 0.030 |
| Cerebrospinal fluid pressure ≥ 180mmH_2_O | 10.90(2.48-47.96) | 0.002 |
| Cerebrospinal fluid pressure ≥ 400mmH_2_O | 3.01(1.12-8.10`) | 0.030 |
| White blood cell abnormality in CSF | 0.83(0.33-2.10) | 0.687 |
| Total protein abnormality in CSF | 0.91(0.36-2.28) | 0.842 |
| Abnormal imaging | 4.37(1.60-11.93) | 0.004 |
| Abnormal electroencephalogram | 4.50(1.70-11.92) | 0.002 |
| Causative agent | | |
| Viral | 1 [Reference] |  |
| Bacterial | 1.82(0.60-5.53) | 0.294 |
| Fungal | 4.82(1.10-21.09) | 0.037 |
| Tubercular | 1.61(0.33-7.92) | 0.560 |

Table 6.Univariate analysis of poor prognosis prediction for severe infectious encephalitis in the ICU

| Variables | OR (95% CI) | *P* |
| --- | --- | --- |
| Mean age (years) | 1.02(1.00-1.04) | 0.032 |
| Gender | 1.24(0.69-2.21) | 0.476 |
| Onset time (days) | 1.06(1.00-1.12) | 0.055 |
| Hospitalized days | 1.01(1.00-1.02) | 0.052 |
| Admission to ICU (days) | 1.03(1.01-1.06) | 0.009 |
| Days of critical condition | 1.03(1.01-1.05) | 0.013 |
| Number of rescuing | 1.15(1.01-1.31) | 0.041 |
| Days of advanced care | 1.05(1.02-1.08) | 0.001 |
| Days of primary care | 1.01(1.00-1.02) | 0.136 |
| Cerebrospinal fluid pressure ≥ 180mmH_2_O | 1.74(0.98-3.09) | 0.057 |
| Cerebrospinal fluid pressure ≥ 400mmH_2_O | 0.89(0.88-4.03) | 0.102 |
| White blood cell in CSF≥10/ul | 1.03(0.57-1.86) | 0.932 |
| White blood cell in CSF≥500/ul | 0.34(0.13-0.95) | 0.039 |
| Total protein in CSF＞0.4g/L | 0.90(0.50-1.63) | 0.930 |
| Total protein in CSF＞1g/L | 1.76(0.96-3.22) | 0.068 |
| Abnormal imaging | 2.48(1.37-4.49) | 0.003 |
| Abnormal electroencephalogram | 1.14(0.62-2.11) | 0.668 |
| Causative agent | | |
| Viral | 1 [Reference] |  |
| Bacterial | 0.94(0.43-2.06) | 0.870 |
| Fungal | 4.96(1.23-20.06) | 0.024 |
| Tubercular | 9.22(2.51-33.91) | 0.001 |
